# Characterization of the Components and Pharmacological Effects of Mountain-Cultivated Ginseng and Garden Ginseng Based on the Integrative Pharmacology Strategy

**DOI:** 10.3389/fphar.2021.659954

**Published:** 2021-04-26

**Authors:** Sen Li, Ping Wang, Wenzhi Yang, Chunhui Zhao, Luoqi Zhang, Jingbo Zhang, Yuewen Qin, Haiyu Xu, Luqi Huang

**Affiliations:** ^1^ College of Chinese Medicinal Materials, Jilin Agricultural University, Changchun, China; ^2^ National Resource Center for Chinese Materia Medica, China Academy of Chinese Medical Sciences, Beijing, China; ^3^ Institute of Chinese Materia Medica, China Academy of Chinese Medical SciencesBeijing, China; ^4^ Tianjin State Key Laboratory of Modern Chinese Medicine, Tianjin University of Traditional Chinese Medicine, Tianjin, China; ^5^ College of Pharmacy, Heilongjiang University of Chinese Medicine, Harbin, China

**Keywords:** mountain-cultivated ginseng, garden ginseng, UHPLC/IM-QTOF-HDMSE, metabolomics analysis, TCMIP V2.0

## Abstract

Panax ginseng C. A. Mey (PGCAM) is a herbaceous perennial belonging to the *Araliaceae* family, mainly including Mountain-Cultivated Ginseng (MCG) and Garden Ginseng (GG) on the market. We aimed to establish a rapid, accurate and effective method to distinguish 15-year-old MCG and GG using ultra-performance liquid chromatography-quadrupole time-of-flight-tandem mass spectrometry (UPLC-QTOF-MS/MS), and also explored the pharmacological mechanisms of the main components using the Integrative Pharmacology-based Network Computational Research Platform of Traditional Chinese Medicine (TCMIP V2.0; http://www.tcmip.cn/). Altogether, 23 potential quality markers were characterized to distinguish 15-year-old MCG and GG, including ginsenosides Ra2, Rg1, and Ra1, and malonyl-ginsenoside Ra3, etc. The contents of 19 constituents (mainly protopanaxadiol-type) were higher in MCG compared with that in GG, and four constituents (mainly carbohydrate compounds) were higher in GG. The 105 putative targets corresponding to 23 potential quality markers were mainly involved in 30 pathways, which could be divided into 10 models, such as immune regulation, systems (metabolic, nervous, cardiovascular, reproductive), blood-pressure regulation, as well as antitumor, antiaging, antibacterial and anti-inflammatory effects. Furthermore, the potential quality markers of MCG and GG could inhibit the proliferation of breast cancer by regulating the mRNA expression of PSA, S6K, MDM2, and P53 genes by acting on AR, MTOR, PI3K and other targets. The Integrative Pharmacology Strategy may provide an efficient way to identify chemical constituents and explore the pharmacological actions of TCM formulations.

## Introduction

Panax ginseng C. A. Mey (PGCAM) is an herbaceous perennial belonging to the *Araliaceae* family. Its dried root and rhizome (Panax ginseng C. A. Mey*.* Radix et Rhizoma) have been used as a herbal medicine in China and some Asian countries for thousands of years (Mancuso and Santangelo, 2017), and have immunomodulation, antifatigue, antiaging, and anticancer effects (Shi et al., 2019). PGCAM was firstly recorded in Shennong’s Classic of Materia Medica (Liu et al., 2013), during which all PGCAM grew wild. However, with the development of society, wild-growing PGCAM could not meet human requirements. To solve the problem, farmers began to study and explore suitable cultivation methods for PGCAM. There are four types of PGCAM according to different cultivation methods: wild-growing ginseng, transplanted ginseng, mountain-cultivated ginseng (MCG) and garden ginseng (GG) (Yang et al., 2013). MCG and GG are recorded in the Pharmacopoeia of the People’s Republic of China from 2005 edition. MCG and GG have become the main varieties on the market.

MCG refers to the seeds of PGCAM germinated and grown in tall mountains and dense forests for 10–20 years (Zhu et al., 2018), so also called “Lin-Xia-Shan-Shen”. If MCG grows long enough, its quality and efficacy become almost the same as that of wild PGCAM. GG refers to PGCAM that planted in a garden and harvested after 4–6 years. In general, the quality of MCG is higher than that of GG, which has a stronger pharmacological effect (Kim et al., 2020). The price of PGCAM is directly proportional to the grown period: the longer the period, the higher the price. Therefore, adulteration or falsification of PGCAM has always been a serious problem in the commercial market.

To solve this problem, researchers have proposed different identification strategies for PGCAM. With the continuous development of new technologies, ultra-performance liquid chromatography-quadrupole time-of-flight-tandem mass spectrometry (UPLC-QTOF-MS/MS) has been widely used in the analysis of chemical components of PGCAM (Wu et al., 2018), multiple compounds have been identified, including ginsenosides, polysaccharides, fatty acids, volatile oils and amino acids (Liang et al., 2016). Ginsenosides, as the main components of PGCAM, show important pharmacological activities during the treatment of cardiovascular diseases (Kim, 2018). In addition, Prof. Xu and his colleague selected 12 chemical components to distinguish GG_4–7 years_ and MCG_15 years_: ginsenoside Ra3/isomer, gypenoside XVII, quinquenoside R1, ginsenoside Ra7, notoginsenoside Fe, ginsenoside Ra2, ginsenoside Rs6/Rs7, malonyl ginsenoside Rc, malonyl ginsenoside Rb1, malonyl ginsenoside Rb2, palmitoleic acid, and ethyl linoleate (Xu et al., 2016). However, the characteristic components and pharmacological effects of MCG and GG grown for 15 years have not been reported. Therefore, we aimed to employ the Integrative Pharmacology Strategy to characterize the differential component between the two types of *Ginseng* and explore their pharmacological effects *in vitro.* Integrative Pharmacology Strategy (IPS) pays attention to the interactions between Chinese prescriptions and the organism from multiple levels and multiple aspects, its research content mainly includes component analysis, network pharmacology analysis and pharmacological experiment verification (Xu and Yang, 2014). The IPS systematically analyzes the interaction between TCM formulations and the organism from multiple levels and multiple aspects to form a new mode of research of TCM. To practice this strategy better, we established Integrative Pharmacology-based Network Computational Research Platform of Traditional Chinese Medicine (TCMIP V2.0; www.tcmip.cn/), which comprising five databases and seven functional modules (Xu et al., 2019).

Therefore, we aimed to identify potential quality markers to distinguish 15-year-old MCG and GG using the UPLC-QTOF-MS/MS. Then, we employed the TCMIP V2.0 to carry out the network pharmacology analysis of the differential components contained in MCG and GG. Finally, we verified the results of network pharmacology *in vitro*. Our research will be a good application of Integrative Pharmacology Strategy.

## Materials and Methods

### Chemicals and Reagents

Twelve batches of MCG and GG were collected from the cultivation areas in Huanren County (41.26°N, 125.36°E; Benxi City, Liaoning Province, China) in September 2017, and the voucher specimens were deposited in our lab. Sixty-six standards of PGCAM were purchased from Shanghai Standard Biotech (Shanghai, China) or isolated from the roots of PGCAM and *Panax notoginseng* C. A. Mey (Yang et al., 2016b). (Supplementary Table S1). HPLC-grade acetonitrile (CH_3_CN) and methanol were purchased from Thermo Fisher Scientific (Fair Lawn, NJ, United States). Formic acid (FA) was obtained from Sigma-Aldrich (Saint Louis, MO, United States). Ultra-pure water was prepared in-house using the Milli-Q™ system (Millipore, Bedford. MA, United States).

### Sample Preparation

First, 50 mg of MCG powder and GG powder were soaked with 3 ml of 70% methanol (v/v), respectively, followed by ultrasound extraction for 60 min at 25°C. Then, the solution was centrifugated at 14,000 rpm for 10 min at room temperature after compensating with 70% methanol for the weight lost. The supernatant was transferred to a 5 ml volumetric flask and diluted to the scale mark. Finally, 1 mlwell-mixed liquid was centrifuged for 10 min, and supernatant were transferred to autosampler vials for analyses. Herbal samples were injected randomly. An equal volume of all test solutions was mixed to prepare the quality control (QC) sample, which was used to monitor the stability of the analytical system.

### Chromatographic Separation and MS Conditions

UPLC-MS was performed on an Acquity™ UPLC I-Class/Vion Ion Mobility Spectrometry (IMS)-QTOF system (Waters, Milford, MA, United States). The chromatographic separation was carried out on a BEH Shield RP18 column (2.1 × 100 mm, 1.7 µm) hyphenated with a VanGuard™ pre-column (2.1 × 50 mm, 1.7 µm; Waters) maintained at 35°C. The mobile phase consisted of 0.1% FA in CH_3_CN (A) and 0.1% FA in H_2_O (B). The optimized gradient program was: 0–2 min, 15–20% A; 2–7 min, 20–30% A; 7–17 min, 30–33% A; 17–20 min, 33–60% A; 20–22 min, 60–98% A; 22–24 min, 98–98% A. The flow rate was 0.3 ml/min and the injection volume was 3 μL. A 3-min re-equilibration time was set between successive injections. A “purge–wash–purge” cycle was set on the autosampler, with 10% CH_3_CN-H_2_O (v/v) as the purge solvent and 50% CH_3_CN-H_2_O as the wash solvent, to minimize the carry-over between injections.

The MS experiment performed on a Vion IMS-QTOF mass spectrometer in the negative electrospray ionization mode (Waters, Corp., Milford, United States). The LockSpray™ ion source was equipped under the following parameters: capillary voltage, 1.0 kV; cone voltage, 20 V; source offset, 80 V; source temperature, 120°C; desolvation gas temperature, 500°C; desolvation gas flow (N_2_), 800 L/h; cone gas flow (N_2_), 50 L/h. Default parameters were defined for the traveling-wave IMS separation. HDMSE data covered a m/z of 300–1,500 at 0.3 s per scan. The low collision energy was set at 6 eV and the high-energy ramp was 40–80 eV. Data acquisition was controlled by the UNIFI 1.9.3.0 software (Waters, Corp., Milford, United States). The accuracy error threshold was fixed at 10 ppm.

### Date Processing

Raw HDMS^E^ data were corrected with reference to m/z 554.2620 in the 12 batches of samples and QC by UNIFI 1.9.3.0. Then, preliminary data were processed automatically by Progenesis QI 2.1 software (Waters, Corp., Milford, CT, United States) [M-H]^−^ and [M + FA-H]^−^ were the main adduct ions in the negative mode. Efficient menu-guided processing, peak alignment and peak selection could generate a data matrix, including retention time (t_R_), m/z, normalized peak area, and collisioncross-secions (CCS). All the ion signals detected in each sample were normalized to the obtained value of the total ion count. The data matrix was filtered based on “80% rule” and “30% variation” (Li J. et al., 2019).

### The Untargeted Metabolomics Analysis Based on Multivariate Statistical Analysis

Processed data were subjected to principal component analysis (PCA) and orthogonal projections to latent structures discriminant analysis (OPLS-DA) using SIMCA-P 14.1 (Umetrics, Umea, Sweden). Potential quality markers were filtered out according to the variable importance for projection (VIP) values (VIP>1.5) and Student’s t-test (*p* < 0.05), which could be used distinguish between MCG and GG.

### Prediction of Putative Targets of Potential Quality Markers

The mol. formats of potential quality markers were uploaded to TCMIP to predict the putative targets using the TCM target prediction and function analysis module (TTFM) of TCMIP according to the similarity of chemical structures to known drugs on the market. The Tanimoto Score was set at 0.7 (moderate similarity) to select constitute–putative target pairs.

### Pathway Enrichment Analysis

To further explore the biological functions of the putative targets, the pathway–enrichment analyses was undertaken using the database for Annotation, Visualization, and Integrated Discovery (DAVID) v6.8 (https://david.ncifcrf.gov). Pathways with *p* < 0.05 were selected for further analyses.

### Cell Culture

MCF-7 cell line was kindly provided by Prof. Xiujie Wang (Institute of Genetics and Developmental Biology, Chinese Academy of Sciences). The MCF-7 cell line was cultured in DMEM high glucose medium (Thermo, Fair Lawn, NJ, United States) with 1% penicillin/streptomycin (Invitrogen, Carlsbad, CA, United States)and 10% fetal bovine serum (FBS; Sijiqing, Huzhou, Zhejiang, China) at 37°C in humid atmosphere with 5% CO_2_.

### Cell Proliferation Analysis

The extraction of MCG and GG was prepared according to Sample Preparation. Then, the methanol was volatilized and the residue was redissolved with sterile water. After filtered by 0.22 μm filter film, the herbal samples were stored in a refrigerator at 4°C. MCF-7 cell line was seeded in 96-well plates (5×10^3^ cells/well) incubating at 37°C for 24 h. Various concentrations of MCG or GG were added to the wells and then incubated for 48 h. Cell proliferation was evaluated using a Cell Counting Kit-8 (CCK8) assay. The cells treated with various conditions were incubated with 10 μL CCK-8 solution (Dojindo, Kumamoto, Japan) for 90 min. Next the absorbance was measured by a Microplate Reader under 450 nm (Thermo, Fair Lawn, NJ, United States). All experiments were performed in triplicate and repeated four times. Data were presented as mean ± standard deviation (SD). A One-way ANOVA determined whether the results had statistical significance.

### Quantitative Real-Time PCR Analysis

The total RNA was isolated from the cultured cells using Trizol (Tiangen, China). Then, cDNA was synthesized using a ReverTra Ace quantitative polymerase chain reaction (PCR) RT Master Mix Kit with gDNA Remover (TOYOBO Co., OSAKA, Japan) according to the manufacturer’s protocol. PCR product amplification was performed with SYBR Green Master Mix (Juhemei, China) on an Agilent Mx 3000P Real-Time PCR System (Applied Biosystems, CA). Primers were 5′-GAATCATCGGACTC AGGTACATC-3′ and 5′-TCT​GTC​TCA​CTA​ATT​GCT​CTC​CT-3′ for MDM2; 5′-CAG​CAC​ATG​ACG​GAG​GTT​GT-3′ and 5′-TCA​TCC​AAA​TAC​TCC​ACA​CGC-3′ for P53; 5′-GTG​AGG​CAG​GCG​ACT​AAT​CAG-3′ and 5′-GTTCCCGTACC TGTTTGCAG-3′ for PSA; 5′-TTT​GAG​CTA​CTT​CGG​GTA​CTT​GG-3′ and 5′-CGA​TGA​AGG​GAT​GCT​TTA​CTT​CC-3′ for S6K; 5′-CTGGAACGGTGAAGGT GACA-3′ and 5′-AAG​GGA​CTT​CCT​GTA​ACA​ATG​CA-3′ for BETA-ACTIN, and 40 PCR cycles (30 s denaturation at 95°C, 20 s annealing at 60°C, 30 s elongation at 72°C) were run. BETA-ACTIN was used as an internal standard. The relative mRNA levels were determined using the 2^−ΔΔCt^ method.

### Statistical Analyses

All data were analyzed by SPSS 22.0 software (SPSS Inc., Chicago,IL, United States). Figures in this research were drawn with GraphPad Prism 8.0 software (GraphPad Prism, San Diego, CA, United States). The results were shown as mean ± standard deviation (SD). Significant differences between normally distributed gene expression data were determined by one-way analysis of variance (ANOVA). The significant difference was set at *p* < 0.05.

## Results

### Differential Components Between MCG and GG Based on Untargeted Metabolomics

**FIGURE 1:**
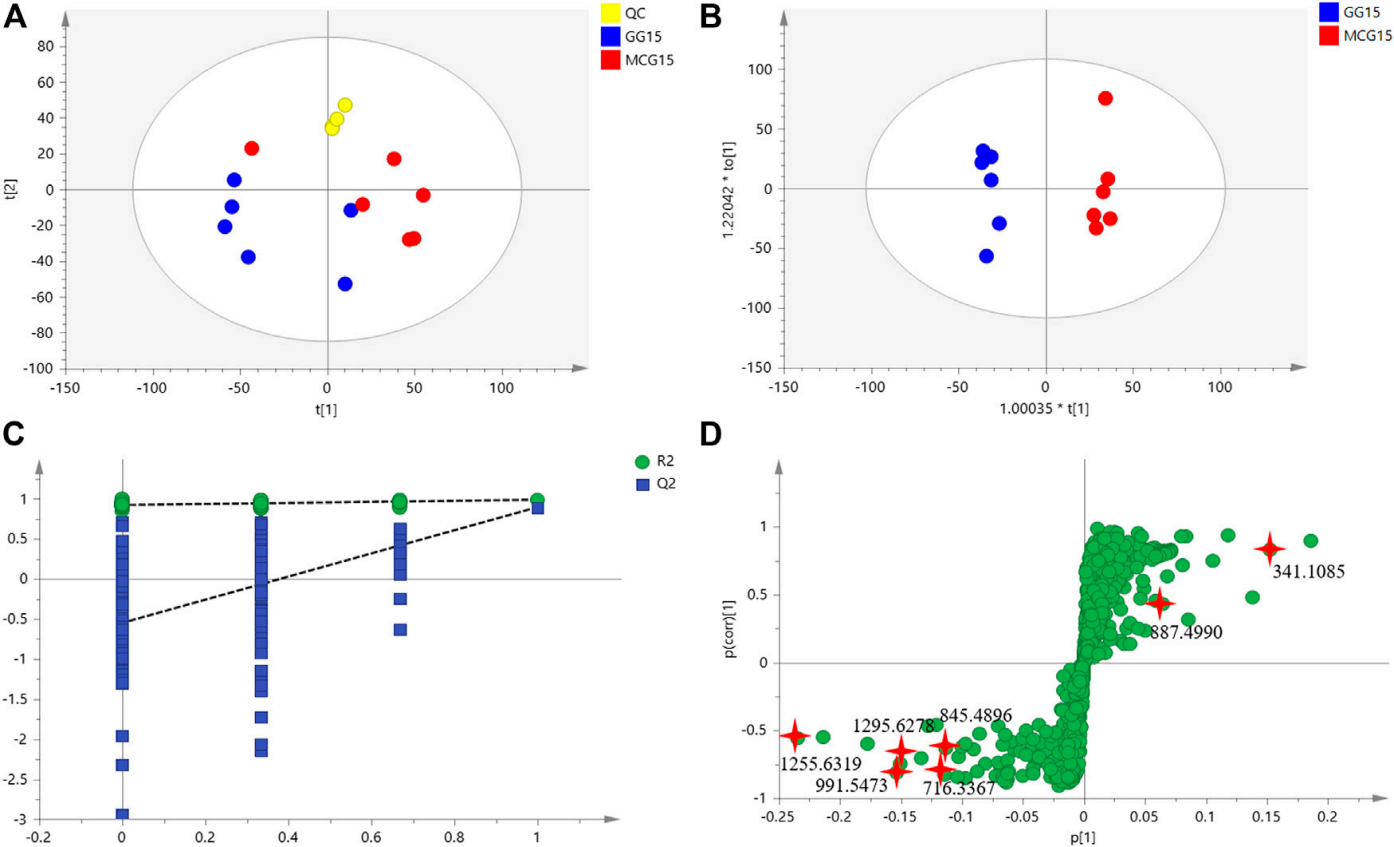
The untargeted metabolomics analysis based on multivariate statistical analysis of MCG and GG. **(A)**: score plot of PCA; **(B)** score plot of OPLS-DA; **(C)** validation plots obtained from 200 permutation tests for the OPLS-DA models; **(D)** score plot of S-Plot.

**FIGURE 2:**
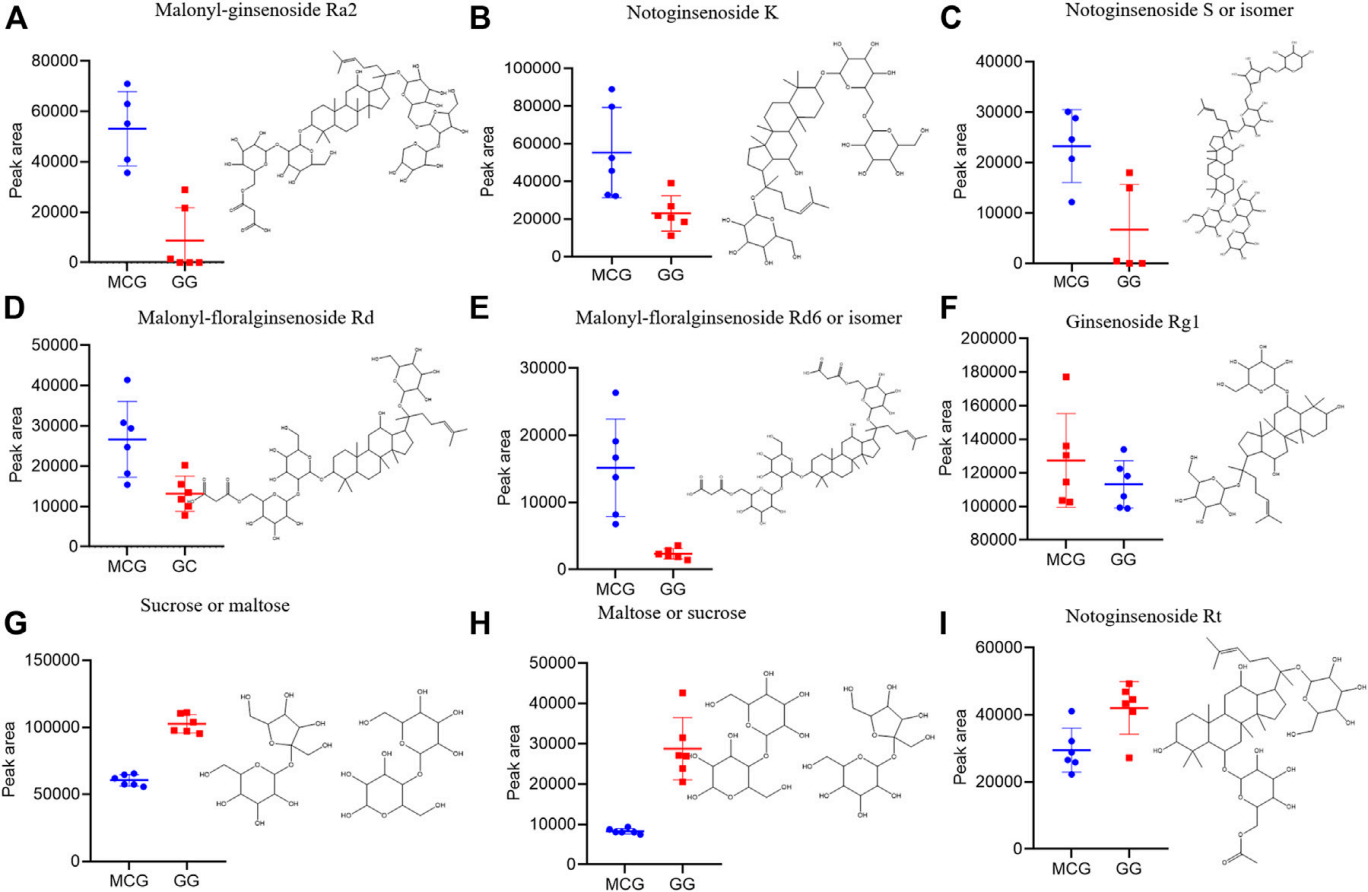
Box charts illustrating the distribution difference of the nine marker compounds among MCG and GG. **(A–F)** can be the characteristic components of MCG. **(G–I)** can be the characteristic components of GG.

Multi-batch HDMS^E^ data were processed by Progenesis QI, and generated a list comprising 1,023 ions (Supplementary Table S2). These ions were filtered based on “80% rule” and “30% variation” (Supplementary Table S3). Then, the remained 954 ions were subjected to PCA and OPLS-DA. MCG and GG groups were separated well in PCA score Plots (Figure1A), indicating the great difference in chemical profiles. To obtain better discrimination, they were subjected to OPLS-DA with R^2^X at 0.865 and Q^2^ at 0.897, especially in the component P1+3 direction (Figure 1B). The validity and predictability of OPLS-DA model was evaluated by 200-time permutation test (Figure 1C). The regression lines of R2 and Q2 decreased with decreased in the correlation coefficients between permuted and original response variables. The result indicated that the predictive OPLS-DA models did not overfit. S-plots were obtained to show responsibility of each ion for these variations more showed intuitively the contribution of differential ions on distinguishing between MCG and GG. Most of the ions clustered around the original point (Figure 1D), and only a few of them were scattered in the margin region. Just these few ions that representing chemical constituents contributed to the separation observed in the score plots. The ions that far away from the original point, always have higher value of VIP (Supplementary Figure S1). Therefore, 42 ions with VIP >1.5 and *p* < 0.05 (in the Student’s t-test) were selected as potential quality markers (Supplementary Table S4), 31 (73.8%) of which were higher in MG, and 11 (26.2%) were higher in GG. Nine representative compounds were illustrated in Figures 2A–I.

### Identification of the Potential Quality Markers

**FIGURE 3:**
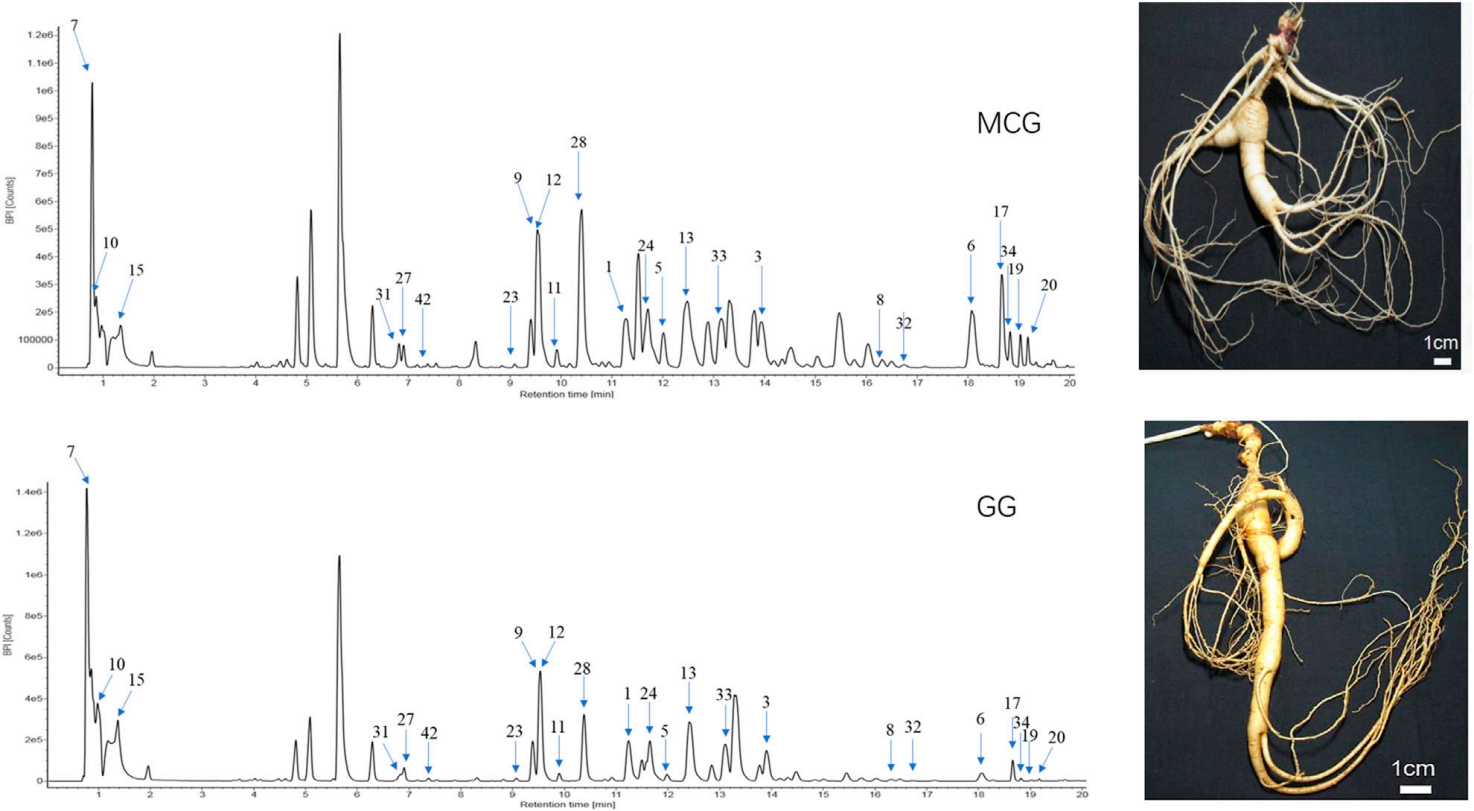
UHPLC/IM-QTOF-HDMS^E^ of MCG and GG in the negative ion modes. MCG: the root and rhizome of Mountain-Cultivated Panax ginseng C. A. Mey; GG: the root and rhizome of Garden Panax ginseng C. A. Mey. (CG).

Sixty-six standard compounds and our house library were helped to identify the 23 potential quality markers. The base peak intensity chromatograms of MCG and GG corresponding to negative ion mode were shown in Figure 3. The 23 identified components with numbers on the peaks were classified as protopanaxadiol (PPD)-type sapogenins (M1, M3, M5, M6, M8, M11, M13, M17, M19, M20, M23, M24, M32, M33, M34, M42), Protopanaxatriol (PPT)-type sapogenins (M9, M12, M27, M28, M31) and carbohydrate compounds (M7, M15). The detailed information of the 23 components was listed in Table 1.

**TABLE 1:** Information of ginsenoside markers for differentiating among MCG and GG.

No	VIP	t_R_ (min)	Measured value (m/z)	Theoretical value (m/z)	Error (ppm)	Formula	Adducts	ESI-MS2	Compound name	References
M1^s^	7.60	11.25	1,255.6319	1,255.6328	−0.7167	C_58_H_98_O_26_	+HCOO	1,209.6271,1077.5858	Ginsenoside Ra2	Zuo et al. (2019)
								945.5293,783.4898		
								621.4375,459.3847		
								375.2879		
M3	5.53	13.92	1,295.6267	1,295.6278	−0.8490	C_61_H_100_O_29_	−H	1,251.6307,1209.6275	Malonyl-ginsenoside Ra1	Yang et al. (2016a)
								1,077.5819,945.5435		
								783.4900,621.4377		
								459.382,375.2831		
M5	4.23	12.00	1,295.6265	1,295.6278	−1.0034	C_61_H_100_O_29_	−H	1,251.6338,1209.6275	Malonyl-ginsenoside Ra2	Yang et al. (2016a)
								1,077.5776,945.5433		
								783.4934,621.4313		
								459.3848,375.2904		
M6^s^	4.11	18.07	991.5473	991.5483	−1.0085	C_48_H_82_O_18_	+HCOO	945.5399,783.4903	Notoginsenoside K	Zuo et al. (2019)
								621.4348,459.4850		
								375.2907		
M7	4.11	0.78	341.1085	341.108	0.000	C_12_H_22_O_11_	−H	—	Sucrose or maltose	—
M8	3.84	16.31	1,295.6282	1,295.6278	0.3087	C_61_H_100_O_29_	+HCOO	1,209.6230,1077.5819	PPD-20-GlcXylXyl-3-GlcGlc-malonyl	Yang et al. (2012)
								945.5395,783.4899		
								621.4408,459.3875		
								375.2977		
M9^s^	3.19	9.54	845.4896	845.4904	−0.9462	C_42_H_72_O_14_	+HCOO	799.4859,637.4303	Ginsenoside Rg1	Li J. et al. (2019)
								475.3788,391.2841		
M11	3.01	9.91	1,325.6375	1,325.6378	−0.2263	C_62_H_102_O_30_	−H	1,107.5964,1077.5855	Malonyl-ginsenoside Ra_3_ or isomer	Yang et al. (2016a), Li J. et al. (2019)
								945.5390,783.4896		
								621.4342,459.3928		
								375.2854		
M12^s^	3.00	9.62	716.3360	716.3367	−0.9772	C_63_H_106_O_30_	+2HCOO	1,341.7149,1209.6689,1077.6205,945.5751	Notoginsenoside S or isomer	—
								783.5146, 637.4337		
M13	2.98	12.35	1,325.6376	1,325.6378	−0.1509	C_62_H_102_O_30_	−H	1,107.5927,1077.5818	Malonyl-ginsenoside Ra_3_ or isomer	Yang et al. (2016a), Li J. et al. (2019)
								945.5395,783.4899		
								621.4408,459.3821		
								375.2929		
M15	2.97	1.35	341.1084	341.1085	−0.2932	C_12_H_22_O_11_	−H	—	Sucrose or maltose	—
M17	2.76	18.66	1,031.5434	1,031.5433	0.0969	C_51_H_84_O_21_	−H	945.5440,783.4870	Malonyl-floralginsenoside Rd	Zuo et al. (2019)
								621.4381,459.3851		
								375.2907		
M19	2.55	19.03	1,117.5429	1,117.5432	−0.2684	C_54_H_86_O_24_	−H	998.4552,945.5363	Malonyl-floralginsenoside Rd6 or isomer	Zhang et al. (2019)
								927.5302,783.4798		
								621.4394,459.3851		
M20	2.34	19.17	1,033.5563	1,033.5583	−1.9351	C_50_H_84_O_19_	+HCOO	987.5576,945.5401	PPD-3GLc-ace	Zhang et al. (2019)
								783.4869,621.4349		
								459.3851,376.9835		
M23	2.16	9.08	731.3414	731.3420	−0.8204	C_64_H_108_O_31_	+2HCOO	621.4376,459.3874	Notoginsenoside T or isomer	—
								375.2953		
M24^s^	2.11	11.61	1,239.6367	1,239.6380	−1.0487	C_59_H_100_O_27_	−H	1,107.5968,1077.5858	Ginsenoside Ra3	Yang et al. (2013)
								945.5432,783.4898		
								621.4376,459.3847		
								375.2,929		
M27^s^	1.93	6.90	887.4990	887.5010	−2.2535	C_44_H_74_O_15_	+HCOO	841.4941,781.4711	Notoginsenoside Rt	
								637.4310,619.4188		
								475.3793,391.2845		
M28	1.91	10.49	887.4999	887.5010	−1.2394	C_44_H_74_O_15_	+HCOO	841.4971,781.4917	Acetyl panajaponol A	Wang et al. (2016)
								637.4240,619.4183		
								475.3789,391.2841		
M31^s^	1.87	6.81	1,031.5432	1,031.5433	−0.0969	C_51_H_84_O_21_	−H	799.4758,637.4341	Malonyl floralginsenoside Re1	Zuo et al. (2019)
								475.3792,391.2869		
M32	1.78	16.74	1,295.6266	1,295.6278	−0.9262	C_61_H_100_O_29_	−H	1,107.5887,1077.5786	PPD-20-GlcXylXyl-3-GlcGlc-malonyl	Yang et al. (2012)
								945.5435,783.4830		
								621.4377,459.3794		
								375.2905		
M33^s^	1.77	13.12	1,255.6302	1,255.6328	−2.0707	C_58_H_98_O_26_	+HCOO	1,209.6275,1077.5862	Ginsenoside Ra1	Zuo et al. (2019)
								945.5396,783.4865		
								621.4377		
M34^s^	1.73	18.82	1,031.5434	1,031.5433	0.0969	C_51_H_84_O_21_	−H	1,077.5992,945.5440	Malonyl-floralginsenoside Rd5	Zuo et al. (2019)
								783.4869,621.4381		
								459.3851,375.2907		
M42	1.51	7.38	971.4854	971.4857	−0.3088	C_48_H_76_O_20_	−H	799.4721,637.4405	(US-5)-glc-glurA-glc	Yang et al. (2012)
								475.3847,391.2894		

s, Ginsenosides identified by comparing with reference standards.

**FIGURE 4:**
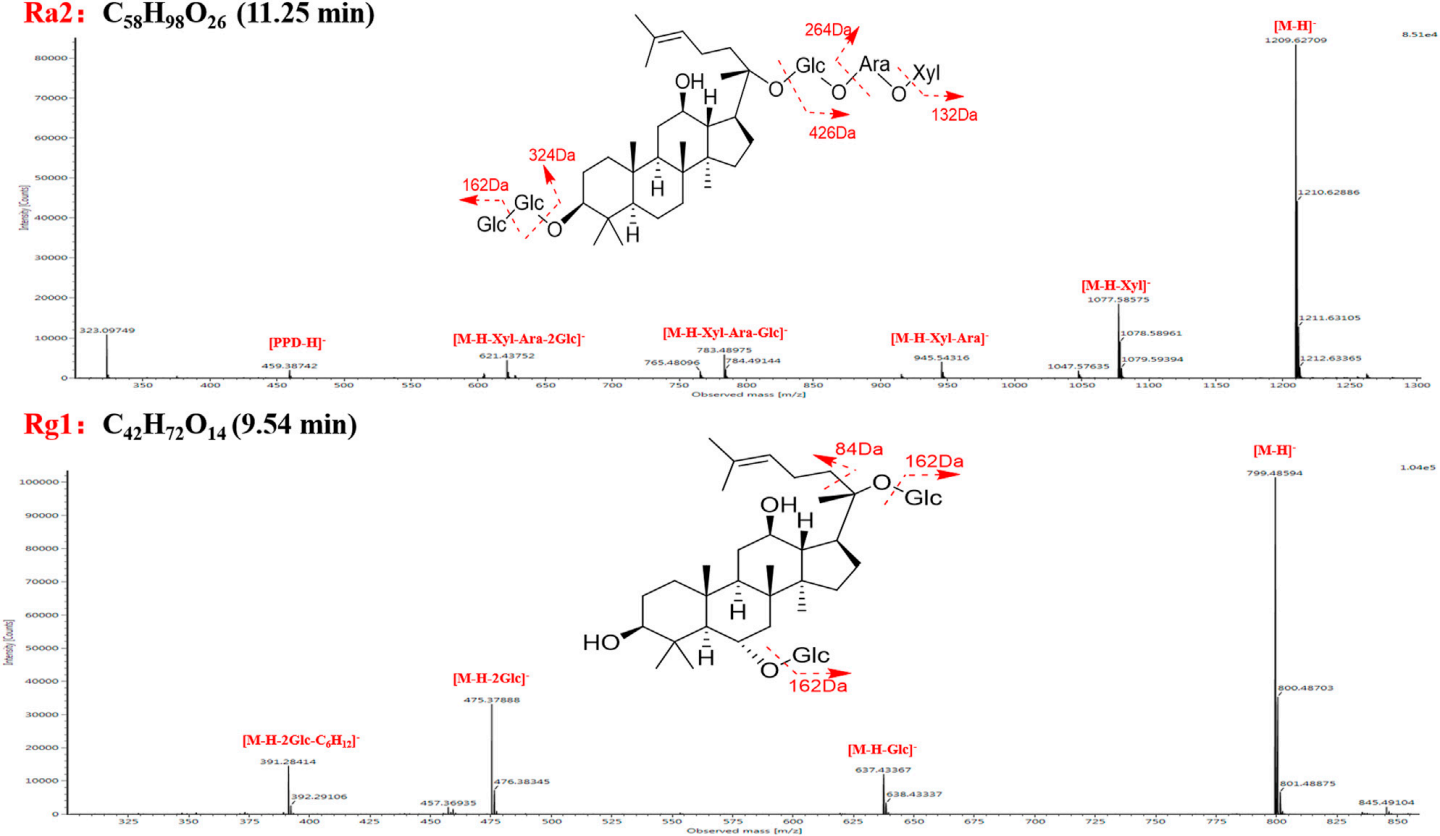
Structural elucidation of ginsenoside Ra2 and Rg1 based on the negative CID-MS^2^ data.

Characteristic MS/MS features of the ginsenoside Ra2 were observed in the MS^2^ spectrum, including m/z 1209.6271 ([M-H]^−^), 1077.5858 ([M-H-Xyl]^−^), 945.5432 ([M-H-Xyl-Ara]^−^), 783.4898 ([M-H-Xyl-Ara-Glc]^−^), 621.4375 ([M-H-Xyl-Ara-2Glc]^−^), and 459.3874 ([PPD-H]^−^) (Figure 4). M1 (ginsenoside Ra2, t_R_, 11.25 min, chemical formula, C_58_H_98_O_26_), M6 (notoginsenoside K, t_R_, 18.07 min, chemical formula, C_48_H_82_O_18_), M24 (ginsenoside Ra3, t_R_, 11.61 min, chemical formula, C_59_H_100_O_27_), M33 (ginsenoside Ra1, t_R_, 13.12 min, chemical formula, C_58_H_98_O_26_), M34 (malonyl-floralginsenoside Rd5, t_R_, 18.82 min, chemical formula, C_51_H_84_O_21_) were identified as PPD-type sapogenins compared with the reference compounds (Supplementary Table S1). Taking ginsenoside Ra2 as an example, the fragmentation pattern of panaxadiol saponins was analyzed.

Taking ginsenoside Rg1 as an example, the fragmentation pattern of panaxatriol saponins was analyzed. Characteristic MS/MS features of ginsenoside Rg1 were observed in the MS^2^ spectrum, including m/z 799.4859 ([M-H]^−^), 637.4334 ([M-H-Glc]^−^), 475.3788 ([M-H-2Glc]^−^), and 391.2841 ([M-H-2Glc-C_6_H_12_]^−^) (Figure 4). M9 (ginsenoside Rg1, t_R_, 9.54 min, chemical formula, C_42_H_72_O_14_), M12 (notoginsenoside S, t_R_, 9.62 min, chemical formula, C_63_H_106_O_30_), M27 (notoginsenoside Rt, t_R_, 6.90 min, chemical formula, C_44_H_74_O_15_), M31 (malonyl-floralginsenoside Re1, t_R_, 6.81 min, chemical formula, C_51_H_84_O_21_) were identified as PPT-type sapogenins compared with the reference compounds (Supplementary Table S1).

### Functional Analysis of Potential Quality Markers

**FIGURE 5:**
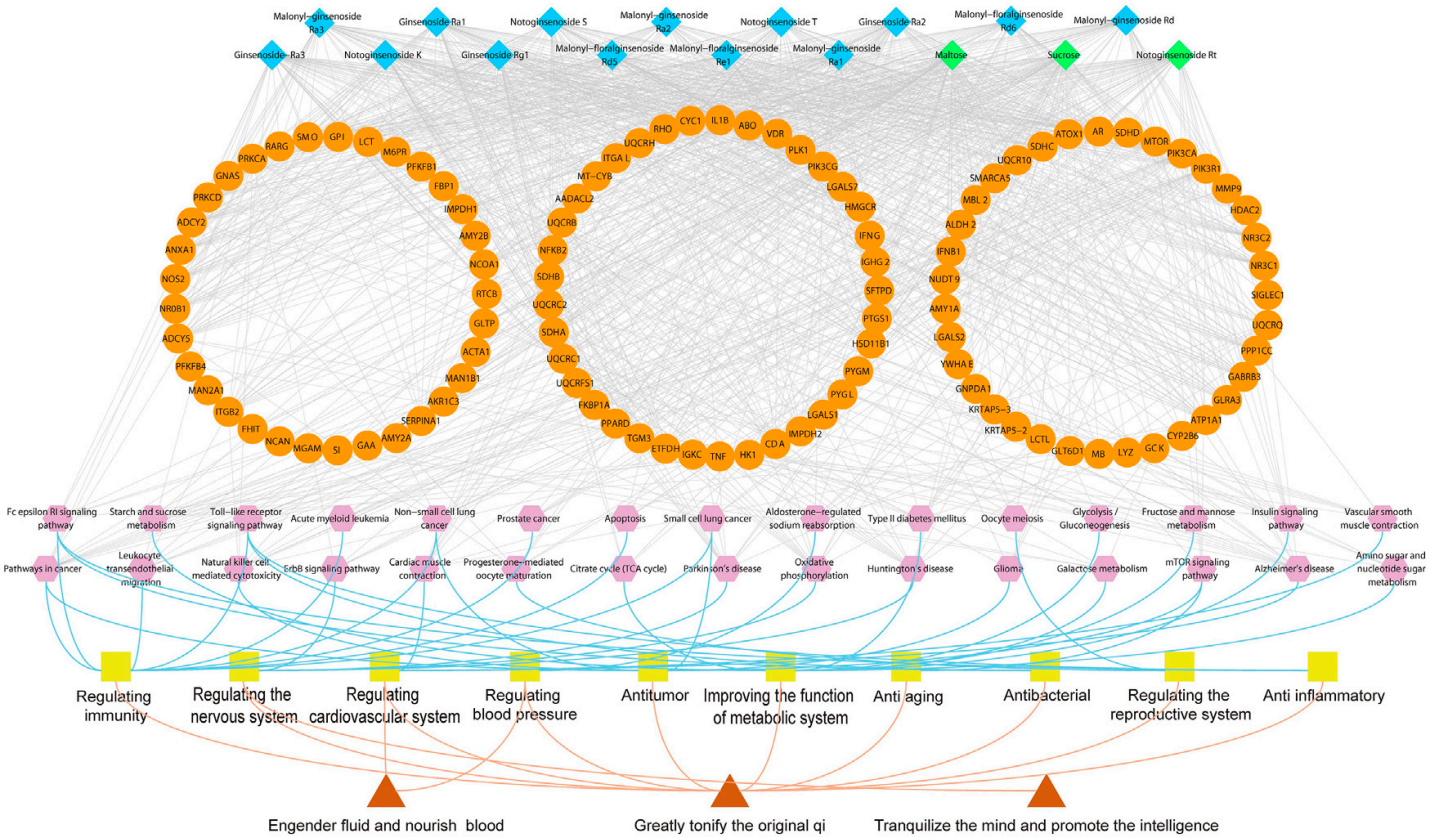
Illustration of the relevance among marker compounds for distinguishing MCG and GG, their target, pathways and therapeutic effects. Blue nodes represent characteristic constituents of MCG. Green nodes represent characteristic constituents of GG. Orange nodes refer to the targets; Purple nodes represent the pathways; Yellow nodes refer to therapeutic effects. Red nodes represent the effect of ginseng.

A total of 105 putative targets were predicated based on 23 primarily identified compounds (Tanimoto score ≥0.7) using TTFM of TCMIP v2.0 (Supplementary Table S5). Which were mainly involved in 30 pathways after functional analysis DAVID 6.8 (Supplementary Table S6). The network of interactions between 23 components, the corresponding 105 putative targets and 30 pathways was visualized using Cytoscape v3.7.1 (Boston, MA, United States) as show in Figure 5. The 30 pathways were related to 10 function modules, such as metabolic system, nervous system, cardiovascular system, immune-regulation system and reproductive system. Among them, there were 12 immune pathways (fc epsilon RI signaling pathway; leukocyte transendothelial migration; non-small cell lung cancer; pathways in cancer; acute myeloid leukemia; toll-like receptor signaling pathway; small cell lung cancer; apoptosis; prostate cancer; natural killer cell mediated cytotoxicity; glioma; mammalian target of rapamycin signaling pathway (mTOR) signaling pathway), nine metabolic pathways (starch and sucrose metabolism; oxidative phosphorylation; type II diabetes mellitus (DM); insulin signaling pathway; galactose metabolism; tricarboxylic acid cycle (TCA cycle); glycolysis/gluconeogenesis; fructose and mannose metabolism; amino sugar and nucleotide sugar metabolism), four nervous system pathways (Alzheimer’s disease; Parkinson’s disease; Huntington’s disease; erbB signaling pathway), there cardiovascular system pathways (cardiac muscle contraction; contraction of vascular smooth muscle; aldosterone-regulated sodium reabsorption) and two reproductive-system pathways (progesterone-mediated oocyte maturation; Oocyte meiosis).

### Inhibitory Effect of MCG and GG on MCF-7 Cell Line by Regulating the PSA, MDM2, P53, and S6K genes

**FIGURE 6:**
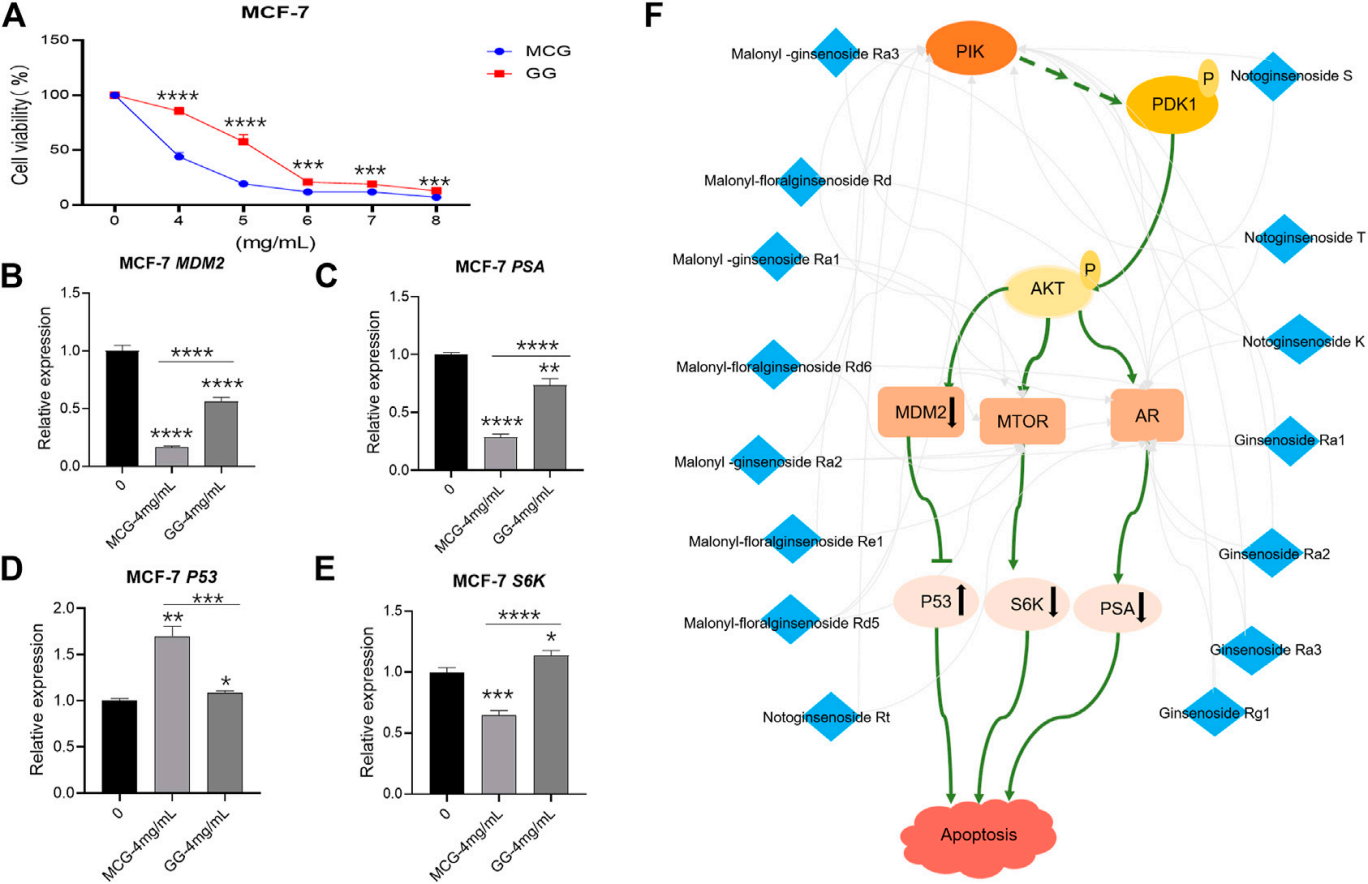
*In vitro* pharmacodynamics experiments and RT-PCR detection of key effector genes in MCG and GG. **(A)**: Results of *in vitro* pharmacodynamics experiments of MCG and GG. **(B–F)**: Results of RT-PCR detection of key effector genes in MCG and GG. “*”: *p* < 0.05; “**”: *p* < 0.01; “***”: *p* < 0.001; “****”: *p* < 0.0001. **(F)**:Mechanism of anti-breast cancer effect of quality markers of MCG.

The enrichment analysis above showed that 23 components played more importment roles in tumor pathway, which indicated that the anticancer effect of MCG was stronger than GG. Therefore, we selected MCF-7 cell line to verify the results. The CCK-8 assay showed that MCG or GG did not significantly inhibit the proliferation of MCF-7 cell line when the concentration was lower than 4 mg/ml. When the concentration of MCG or GG was 8 mg/ml, the survival rate of MCG group was only 11.8%, and GG group was 21.99%, which indicated that the concentration of 4–8 mg/ml was the range of MCG and GG to inhibit the proliferation of MCF-7 cell line (Supplementary Figure S2). Thus, the concentrations of 4, 5, 6, 7, and 8 mg/ml were set to explore the inhibitory effect of MCG and GG on MCF-7 cell line. When the extract concentration was 4 mg/ml, the survival rate of MCF-7 cell line was significantly inhibited by MCG compared with GG (Figure 6A).

Therefore, MG and GG at 4 mg/ml were selected to subject to RT-PCR detection. The mRNA expression of PSA, MDM2 and S6K genes in the group of MCG was significantly down-regulated compared with that of control group, and the degree of down regulation was stronger than that of GG. Conversely, the mRNA expression of *P53* gene was significantly up-regulated, and the degree stronger than that of GG (Figures 6B–E).

## Discussion

PGCAM as a herbal nutritional supplement is well known for its extensive pharmacological effects (Nguyen and Nguyen, 2019). MCG and GG are the main PGCAM varieties on the market to meet the huge demand of people (Yang et al., 2013) (Shen et al., 2019). At present, ginsenoside is the main marker for identification of different species of MCG and GG. For example, ginsenosides can be used to distinguish different planting methods and different years of ginseng (Cui et al., 2013; Ruan et al., 2018). As natural plants, the environment and duration of growth affect the quality and price of PGCAM directly. (Xu et al., 2016; Zhu et al., 2018). Many studies have been undertaken to characterize the chemical profiles of PGCAM cultivated in different environments for various durations, which could help distinguish diverse samples (Chang et al., 2016; Chang et al., 2017). Hence, we aimed to employ integrative pharmacology strategy to characterize the differential components between the two types of *Ginseng* and explore their pharmacological effects by network pharmacology and verified *in vitro.*

Altogether, 23 chemical components were identified to distinguish the two sets of samples, and nine of them were identified according to use of reference compounds. The characteristic fragments of PPD-saponins were m/z 459.38 and m/z 375.29. These data could be used to identify unknown marker compounds belonging to this type, including M14, M16, M18, M26, M29, M30, M36, M39, and M41. Ginsenosides were the main characteristic components of the MCG group. Carbohydrates were the main characteristic components of the GG group. The peak area of PPD-type saponins in the MCG group was higher than that of GG group, and the peak area of carbohydrate compounds was higher in the GG group. The results showed that ginsenoside Ra2 and sucrose could not only distinguish 15 years old MCG and GG, but also 10–20 years old MCG and 4–6 years old GG (Xu et al., 2016). Ginsenoside Rg1 can not only distinguish the 15-year-old forest ginseng and garden ginseng, but also distinguish the ginseng of different ages, different planting sites and different slope directions (Zhu et al., 2021). In conclusion, some of the quality markers in the same region can distinguish ginseng under different conditions. It shows that our research results have certain universality.

To explore further the pharmacological activities of these marker components, components–targets–pathways network was constructed. We found that the marker components could regulate immune, metabolic, nervous–system, cardiovascular–system and reproductive–system pathways. Among them, immunomodulatory and metabolic regulatory pathways were the main pathways regulated by marker components during the treatment of cancer and diabetes mellitus.

The pharmacological effects of marker components were investigated further. Ginsenoside Rg1 has been found to: 1) protect the heart from cardiovascular diseases (Xu et al., 2018); 2) impact the neuroendocrine system for treatment of depression (Mou et al., 2017); 3) inhibit inflammation and apoptosis (Guo et al., 2019); 4) possess neuroprotective properties (Li G. et al., 2019); 5) enhance gene expression and oxidative muscle metabolism in muscles (Jeong et al., 2019); 6) protect against diabetic nephropathy (DN) by reducing oxidative stress (Du et al., 2018). Hence, Rg1 can regulate the cardiovascular system, nervous system and immune system. Sucrose can reduce procedural pain from single events, including heel lancing, venipuncture and intramuscular injection, which shows that sucrose can regulate the nervous system without side effects. (Stevens et al., 2016). Ginsenoside Ra1 is the main active component of ginseng used for immune regulation (Liu et al., 2019). Hence, ginsenosides Ra1 can affect the cardiovascular system and regulate the immune system. Notoginsenoside K has been shown to exhibit immunologic-adjuvant activities on the humoral immune responses of ICR mice against ovalbumin (Sun et al., 2005; Qin et al., 2006). Ginsenosides Ra1, notoginsenoside K, and ginsenoside Rg1 can affect the cardiovascular system, immune regulation, nervous system, and have anti-inflammatory and antioxidant effects. Sucrose has a protective effect upon HepG2 cells and fibroblasts (Sim et al., 2020), which suggests that sucrose has an antioxidant effect. Maltose can enhance the anti-melanogenic activity (Bin et al., 2017). The results showed that the pharmacological activities of potential quality markers were consistent with those reported in the literature. Seven malonylginsenosides (malonyl-ginsenoside Ra1, malonyl-ginsenoside Ra2, malonyl-ginsenoside Ra3, malonyl-floralginsenoside Rd, malonyl-floralginsenoside Rd6, malonyl-floralginsenoside Re1 and malonyl-floralginsenoside Rd5) were identified as the PPD-type markers of MCG and GG. However, reports on the pharmacologic effects and mechanism of action of malonyl saponins are lacking, which needed further study.

Network pharmacology pathway enrichment showed that the cancer pathways enriched by quality markers were more abundant. Therefore, we used MCF-7 cell line to evaluate the anticancer effect of MCG and GG. The results showed that the anticancer effect of MCG was better than GG, which was consistent with the published data (Kim et al., 2020). The main genes involve in anticancer effect of MCG and GG are PIK3CA, PIK3CG, MTOR and AR, which are also the key genes of PI3K/Akt/mTOR pathway. Among them, the quality markers acting on PIK3CA, PIK3CG and AR were malonyl -ginsenoside Ra3, malonyl-floralginsenoside Rd, malonyl -ginsenoside Ra1, malonyl-floralginsenoside Rd6, malonyl -ginsenoside Ra2, malonyl-floralginsenoside Re1, malonyl-floralginsenoside Rd5, notoginsenoside Rt, notoginsenoside S, notoginsenoside T, notoginsenoside K, ginsenoside Ra1, ginsenoside Ra2, ginsenoside Ra3, Ginsenoside Rg1. The potential quality markers acting on MTOR were malonyl -ginsenoside Ra3, malonyl-floralginsenoside Rd, malonyl -ginsenoside Ra1, malonyl-floralginsenoside Rd6, malonyl -ginsenoside Ra2, malonyl-floralginsenoside Re1, malonyl-floralginsenoside Rd5, which were common quality markers of PIK3CA, PIK3CG, MTOR. Except Notoginsenoside Rt, the content of the remaining 14 quality markers in MCG was higher than GG. In conclusion, the 14 quality markers of MCG play a better role in anticancer by acting on PI3K/Akt/mTOR pathway while the data of mRNA expression of PIK3CA, PIK3CG, MTOR, AR in the MCF-7 cell samples treated by MCG and GG showed that there was no significant difference between ginseng treatment group and control group. However, we found that downstream genes of the PI3K pathway play an important role in cancer therapy through literature research. In addition, MDM2 has been shown to be abnormally upregulated due to gene amplification, increased transcription, and enhanced translation, leading to enhanced degradation and reduction of P53 activity in some human tumors (Konopleva et al., 2020)and therefore, many drugs/compounds have been developed for reactivation of P53 gene by inhibiting MDM2 interaction with P53 in order to treat cancer (Gupta et al., 2019). Moreover, MTOR pathway plays an important role in the treatment of human cancer. mTORC1 mediates its function via its downstream targets 40 S ribosomal S6 kinases (S6K) (Sridharan and Basu, 2020). Prostate specific antigen (PSA) is a serine protease produced by prostate epithelial cells and prostate cancer (PCA), which can be regulated by AR. The increase of PSA level can be used as a diagnostic marker of tumor or cancer (Balk et al., 2003). The RT-PCR data showed that mRNA expression of MDM2, S6K, PSA were significantly decreased, and the mRNA expression of P53 gene was increased in MCG compared with GG. In summary, the results show that the quality markers of MCG can reduce the survival rate of breast cancer cells better by acting on PIK3CG, PIK3CA, mTOR, AR genes and co-regulating the effector genes MDM2, P53, PSA, S6K (Figure 6F).

## Conclusion

We characterized, for the first time, 23 chemical components to distinguish 15-year-old MCG and GG, which corresponding to 105 putative targets and 30 pathways. In addition, the 23 components inhibit the proliferation of breast cancer (MCF-7 cells) by regulating the mRNA expression of *PSA*, *S6K*, *MDM2* and *P53*, in which MG exhibited stronger effects than that of GG. Integrative Pharmacology Strategy undoubtedly provide an efficient way to identify chemical constituents and explore the pharmacological actions of TCM formulations.

## Data Availability

The original contributions presented in the study are included in the article/Supplementary Material, further inquiries can be directed to the corresponding authors.
