# Supplementary material for: Characterization of the Components and Pharmacological Effects of Mountain-Cultivated Ginseng and Garden Ginseng Based on the Integrative Pharmacology Strategy
Source: Front Pharmacol. 2021 Apr 26;12:659954. doi: 10.3389/fphar.2021.659954 (PMC8108004; doi:10.3389/fphar.2021.659954)
Supplement: Supplementary file 3 [file Table1.DOCX]

Table S1 Information of 66 ginsenoside reference compounds used this work

| NO. | 标准品 | 分子式 | 分子量 | Sub Class |
| --- | --- | --- | --- | --- |
| 1 | 20s-protopanaxatriol | [C_30_H_52_O_4_](https://pubchem.ncbi.nlm.nih.gov/#query=C30H52O4) | 476.3866 | PPD |
| 2 | Protopanaxatriol | [C_30_H_52_O_4_](https://pubchem.ncbi.nlm.nih.gov/#query=C30H52O4) | 476.3866 |  |
| 3 | Pseudoginsenoside Rh2 | [C_36_H_62_O_8_](https://pubchem.ncbi.nlm.nih.gov/#query=C36H62O8) | 622.4445 |  |
| 4 | Ginsenoside Rh2 | [C_36_H_62_O_8_](https://pubchem.ncbi.nlm.nih.gov/#query=C36H62O8) | 622.4445 |  |
| 5 | 20R- ginsenoside Rh2 | [C_36_H_62_O_8_](https://pubchem.ncbi.nlm.nih.gov/#query=C36H62O8) | 622.4445 |  |
| 6 | 20s- ginsenoside Rh2 | [C_36_H_62_O_8_](https://pubchem.ncbi.nlm.nih.gov/#query=C36H62O8) | 622.4445 |  |
| 7 | Ginsenoside Rg3 | [C_42_H_72_O_13_](https://pubchem.ncbi.nlm.nih.gov/#query=C42H72O13) | 784.4973 |  |
| 8 | 20R- ginsenoside Rg3 | [C_42_H_72_O_13_](https://pubchem.ncbi.nlm.nih.gov/#query=C42H72O13) | 784.4973 |  |
| 9 | Ginsenoside F2 | [C_42_H_72_O_13_](https://pubchem.ncbi.nlm.nih.gov/#query=C42H72O13) | 784.4973 |  |
| 10 | Ginsenoside RS3 | [C_44_H_74_O_14_](https://pubchem.ncbi.nlm.nih.gov/#query=C44H74O14) | 826.5079 |  |
| 11 | Ginsenoside Rd2 | [C_47_H_80_O_17_](https://pubchem.ncbi.nlm.nih.gov/#query=C47H80O17) | 916.5396 |  |
| 12 | Notoginsenoside Fd | [C_47_H_80_O_17_](https://pubchem.ncbi.nlm.nih.gov/#query=C47H80O17) | 916.5396 |  |
| 13 | Notoginsenoside K | [C_48_H_82_O_18_](https://pubchem.ncbi.nlm.nih.gov/#query=C48H82O18) | 946.5501 |  |
| 14 | Ginsenoside Rd | [C_48_H_82_O_18_](https://pubchem.ncbi.nlm.nih.gov/#query=C48H82O19) | 946.5501 |  |
| 15 | Malonyl-floralginsenoside Rd5 | C_51_H_84_O_21_ | 1032.5505 |  |
| 16 | Ginsenoside Rb2 | [C_53_H_90_O_22_](https://pubchem.ncbi.nlm.nih.gov/#query=C53H90O22) | 1078.5924 |  |
| 17 | Ginsenoside Rb3 | [C_53_H_90_O_22_](https://pubchem.ncbi.nlm.nih.gov/#query=C53H90O22) | 1078.5924 |  |
| 18 | Ginsenoside Rc | [C_53_H_90_O_22_](https://pubchem.ncbi.nlm.nih.gov/#query=C53H90O22) | 1078.5924 |  |
| 19 | Ginsenoside Rb1 | [C_54_H_92_O_23_](https://pubchem.ncbi.nlm.nih.gov/#query=C54H92O23) | 1108.6029 |  |
| 20 | Malonyl-ginsenoside Rc | C_56_H_92_O_25_ | 1164.5928 |  |
| 21 | Malonyl-ginsenoside Rb2 | C_56_H_92_O_25_ | 1164.5928 |  |
| 22 | Ginsenoside Ra2 | [C_58_H_98_O_26_](https://pubchem.ncbi.nlm.nih.gov/#query=C58H98O26) | 1210.6346 |  |
| 23 | Ginsenoside Ra1 | [C_58_H_98_O_26_](https://pubchem.ncbi.nlm.nih.gov/#query=C58H98O26) | 1210.6346 |  |
| 24 | Notoginsenoside R4 | [C_59_H_100_O_27_](https://pubchem.ncbi.nlm.nih.gov/#query=C59H100O27) | 1240.6452 |  |
| 25 | Ginsenoside Ra3 | [C_59_H_100_O_27_](https://pubchem.ncbi.nlm.nih.gov/#query=C59H100O27) | 1240.6452 |  |
| 26 | Notoginsenoside S | [C_63_H_106_O_30_](https://pubchem.ncbi.nlm.nih.gov/#query=C63H106O30) | 1342.6769 |  |
| 27 | Notoginsenoside T | [C_64_H_108_O_31_](https://pubchem.ncbi.nlm.nih.gov/#query=C64H108O31) | 1372.6875 |  |
| 28 | Ginsenoside Rh8 | [C_36_H_60_O_9_](https://pubchem.ncbi.nlm.nih.gov/#query=C36H60O9) | 636.4238 | PPT |
| 29 | Ginsenoside Rh7 | [C_36_H_60_O_9_](https://pubchem.ncbi.nlm.nih.gov/#query=C36H60O9) | 636.4238 |  |
| 30 | Ginsenoside Rh1 | [C_36_H_60_O_9_](https://pubchem.ncbi.nlm.nih.gov/#query=C36H60O9) | 638.4394 |  |
| 31 | Ginsenoside F1 | [C_36_H_60_O_9_](https://pubchem.ncbi.nlm.nih.gov/#query=C36H60O9) | 638.4394 |  |
| 32 | 20R- ginsenoside Rh1 | [C_36_H_60_O_9_](https://pubchem.ncbi.nlm.nih.gov/#query=C36H60O9) | 638.4394 |  |
| 33 | Ginsenoside F5 | C_41_H_70_O_13_ | 770.4816 |  |
| 34 | 20R-notoginsenoside R2 | [C_41_H_70_O_13_](https://pubchem.ncbi.nlm.nih.gov/#query=C41H70O13) | 770.4816 |  |
| 35 | Notoginsenoside R2 | [C_41_H_70_O_13_](https://pubchem.ncbi.nlm.nih.gov/#query=C41H70O13) | 770.4816 |  |
| 36 | Ginsenoside F3 | [C_41_H_70_O_13_](https://pubchem.ncbi.nlm.nih.gov/#query=C41H70O13) | 770.4816 |  |
| 37 | 20s- Notoginsenoside R2 | [C_41_H_70_O_13_](https://pubchem.ncbi.nlm.nih.gov/#query=C41H70O13) | 770.4816 |  |
| 38 | Pseudoginsenoside Rt3 | [C_41_H_70_O_13_](https://pubchem.ncbi.nlm.nih.gov/#query=C41H70O13) | 770.4816 |  |
| 39 | Ginsenoside Rg2 | [C_42_H_72_O_13_](https://pubchem.ncbi.nlm.nih.gov/#query=C42H72O13) | 784.4973 |  |
| 40 | Ginsenoside Rg1 | [C_42_H_72_O_14_](https://pubchem.ncbi.nlm.nih.gov/#query=C42H72O14) | 800.4922 |  |
| 41 | Ginsenoside Rf | C_42_H_72_O_14_ | 800.4922 |  |
| 42 | Notoginsenoside Rt | C_44_H_74_O_15_ | 842.5028 |  |
| 43 | Notoginsenoside Ft1 | [C_47_H_80_O_17_](https://pubchem.ncbi.nlm.nih.gov/#query=C47H80O17) | 916.5396 |  |
| 44 | Notoginsenoside R1 | [C_47_H_80_O_18_](https://pubchem.ncbi.nlm.nih.gov/#query=C47H80O18) | 932.5345 |  |
| 45 | Notoginsenoside Fp1 | [C_47_H_80_O_18_](https://pubchem.ncbi.nlm.nih.gov/#query=C47H80O18) | 932.5345 |  |
| 46 | Ginsenoside Re3 | C_48_H_82_O_18_ | 946.5501 |  |
| 47 | Ginsenoside Re2 | C_48_H_82_O_18_ | 946.5501 |  |
| 48 | Ginsenoside Re | C_48_H_82_O_18_ | 946.5501 |  |
| 49 | Vinaginsenoside R4 | C_48_H_82_O_19_ | 962.5450 |  |
| 50 | 20-o-glucosylginsenoside Rf | C_48_H_82_O_19_ | 962.5450 |  |
| 51 | Notoginsenoside N | C_48_H_82_O_19_ | 962.5450 |  |
| 52 | Malonyl-floralginsenoside Re1 | C_51_H_84_O_21_ | 1032.5505 |  |
| 53 | Pseudoginsenoside Rt1 | [C_47_H_74_O_18_](https://pubchem.ncbi.nlm.nih.gov/#query=C47H74O18) | 926.4875 | OA |
| 54 | Chikusetsusaponin IV | C_47_H_74_O_18_ | 926.4875 |  |
| 55 | Ginsenoside Ro | [C_48_H_76_O_19_](https://pubchem.ncbi.nlm.nih.gov/#query=C48H76O19) | 956.4981 |  |
| 56 | 24R-pseudoginsenoside-Rt5 | [C_36_H_62_O_10_](https://pubchem.ncbi.nlm.nih.gov/#query=C36H62O10) | 654.4343 | OT |
| 57 | Pseudoginsenoside F11 | C₄₂H₇₂O₁₄ | 800.4922 |  |
| 58 | Ginsenoside Rh3 | [C_36_H_60_O_7_](https://pubchem.ncbi.nlm.nih.gov/#query=C36H60O7) | 604.4338 | Others |
| 59 | Ginsenoside Rk2 | [C_36_H_60_O_7_](https://pubchem.ncbi.nlm.nih.gov/#query=C36H60O7) | 604.4339 |  |
| 60 | Ginsenoside Rh4 | [C_36_H_60_O_8_](https://pubchem.ncbi.nlm.nih.gov/#query=C36H60O8) | 620.4288 |  |
| 61 | Ginsenoside Rk3 | [C_36_H_60_O_8_](https://pubchem.ncbi.nlm.nih.gov/#query=C36H60O8) | 620.4288 |  |
| 62 | Notoginsenoside T5 | [C_41_H_68_O_12_](https://pubchem.ncbi.nlm.nih.gov/#query=C41H68O12) | 752.4711 |  |
| 63 | Ginsenoside Rg6 | [C_42_H_70_O_12_](https://pubchem.ncbi.nlm.nih.gov/#query=C42H70O12) | 766.4868 |  |
| 64 | Ginsenoside Rk1 | [C_42_H_70_O_12_](https://pubchem.ncbi.nlm.nih.gov/#query=C42H70O12) | 766.4868 |  |
| 65 | Ginsenoside Rg5 | [C_42_H_70_O_12_](https://pubchem.ncbi.nlm.nih.gov/#query=C42H70O12) | 766.4868 |  |
| 66 | Ginsenoside F4 | [C_42_H_70_O_12_](https://pubchem.ncbi.nlm.nih.gov/#query=C42H70O12) | 766.4868 |  |
