# Supplementary figures and images for: Characterization of the Components and Pharmacological Effects of Mountain-Cultivated Ginseng and Garden Ginseng Based on the Integrative Pharmacology Strategy
Source: Front Pharmacol. 2021 Apr 26;12:659954. doi: 10.3389/fphar.2021.659954 (PMC8108004; doi:10.3389/fphar.2021.659954)

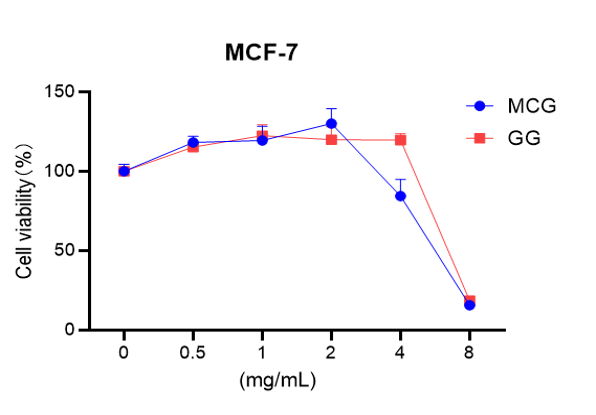

Supplement: Supplementary file 4 [file Image2.TIF]

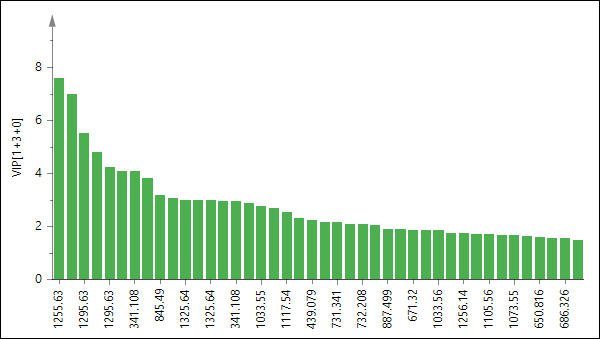

Supplement: Supplementary file 5 [file Image1.TIF]
